# Safety and tolerability of venom immunotherapy: Evaluation of 581 rush- and ultra-rush induction protocols (safety of rush and ultra-rush venom immunotherapy)

**DOI:** 10.1016/j.waojou.2020.100496

**Published:** 2020-12-15

**Authors:** Richard Stock, Tatjana Fischer, Katharina Aẞmus, Nadja Zoeller, Hanns Ackermann, Roland Kaufmann, Markus Meissner, Eva Valesky

**Affiliations:** a Department of Dermatology, Venereology and Allergology, University Hospital, Goethe University of Frankfurt, Theodor Stern Kai 7, D-60590, Frankfurt am Main, Germany; b Institute for Biostatistics, University Hospital, Goethe University of Frankfurt, Theodor Stern Kai 7, D-60590, Frankfurt, Germany

**Keywords:** Hymenoptera venom immunotherapy, Ultra-rush protocol, Rush protocol, Bee venom allergy, Vespid venom allergy, BTC, basal tryptase concentration, BV, bee venom, f, female, m, male, VIT, venom immunotherapy, IgE, Immunoglobulin E, ml, millilitres, μL, microlitres, n, number of patients, R, rush, UR, ultra-rush, y, years, VV, vespid venom

## Abstract

**Background:**

Current literature is inconsistent regarding the risk of severe side effects using accelerated induction protocols in Hymenoptera venom immunotherapy (VIT). In addition, several data indicate the influence of purity grade of venom preparation on tolerability. We evaluated the safety and tolerability of ultra-rush and rush build-up protocols using purified and non-purified venom preparations.

**Methods:**

Retrospective single-center study of 581 VIT inductions (325 ultra-rush and 256 rush protocols) from 2005 to 2018 in 559 patients with bee and vespid venom allergy using aqueous purified (ALK SQ®) for ultra-rush protocol and aqueous non-purified (ALK Reless®) venom preparations for rush protocol.

**Results:**

Urticaria (8% vs. 3.1%, p = 0,013) and dose reductions (4.3% vs. 1.2%, p = 0,026) were significantly more frequent in the ultra-rush group. Overall rate of moderate-to-severe side effects (anaphylaxis ≥grade 2 according to *Ring and Meβmer*) was low and did not differ significantly between protocols (p = 0.105). Severe events (grade 4 anaphylaxis) were not reported. Discontinuation rate was very low in both cohorts (0.6% vs 1.2%). The higher purity grade of venom preparations in the ultra-rush cohort did not improve tolerability. The bee venom group showed a non-significant trend towards higher incidence of mild reactions (urticaria), resulting in more frequent dose reductions and antiallergic therapy.

**Conclusion:**

Rush and ultra-rush protocols show an excellent safety profile with only infrequent and mild anaphylactic reactions in bee and vespid venom allergy. Ultra-rush immunotherapy reduces the duration of the inpatient build-up phase setting and thus is viewed by the authors as preferred treatment in Hymenoptera venom allergic patients.

## Introduction

Insect stings by Hymenoptera species are frequent with up to 94.5% of the general population stung at least once in their lifetime.^1^ The prevalence of sensitization to Hymenoptera venom ranges from 27% to up to 40% among adults and up to 50% among children.^2^ Adults with a high risk of being stung show increased sensitization rates of up to 58% in wasp venom and 50% in honey bee venom.^3^ The majority of these sensitizations remain clinically irrelevant. In Europe, 0.3% to 7.5% of adults and up to 3.4% of children suffer from anaphylaxis due to Hymenoptera venom.^4^ Stings of the vespid species *Vespula vulgaris* and *Vespula germanica* followed by the honeybee *Apis mellifera* are the primary cause of anaphylaxis in adults in Central Europe.^4^

A number of risk factors associated with severe anaphylactic reactions have been identified.^5^ These include older age, male sex, white race, vigorous exercise, as well as cardiovascular disease and concomitant betablocker or ACE-inhibitor intake.^6^, ^7^, ^8^ Asthma remains a controversial risk factor in insect venom anaphylaxis.^8^ Elevated serum tryptase concentrations >11.4 μg/L and patients with systemic mastocytosis also have an increased risk of severe anaphylaxis following Hymenoptera stings.^9^

As the only causal treatment for Hymenoptera venom allergy available to date, venom immunotherapy (VIT) has been shown to be a highly effective and safe therapy.

Based on current European guidelines, VIT is indicated for ≥ grade 2 anaphylactic reactions according to the *Ring and Meßmer* classification^5^^,^^10^ with evidence of IgE-mediated sensitization by skin prick test and/or specific serum IgE against Hymenoptera venom. Immunotherapy is also warranted for grade 1 anaphylaxis (reactions limited to the skin) if patients are at increased risk of exposure or experience impairment in quality of life. Protection from future severe reactions can be achieved in 91%–96% for vespid venom allergy and in 77%–84% for honeybee venom allergy.^2^

The frequency of systemic adverse events with VIT in recent multicenter studies ranges from 8 to 20%.^11^, ^12^, ^13^ In order to improve tolerability and reduce severe side effects, different dosing protocols (conventional, rush, cluster, and ultra-rush protocol) have been established.^14^, ^15^, ^16^, ^17^, ^18^, ^19^ To date, there are only limited data available comparing the safety of rush and ultra-rush protocols with controversial findings regarding the rate of adverse events during immunotherapy and risk factors associated with severe anaphylactic reactions. The conventional, slower induction protocols have been reported to have a higher tolerability compared to the rush and ultra-rush schemes.^4^^,^^20^ Other studies, however, found that two-day ultra-rush protocols were safer than protocols of a longer duration involving a larger number of injections^15^ and show superior tolerability with lower cumulative doses.^21^

In Europe, purified and non-purified aqueous venom extracts are commercially available for VIT build-up (ultra-rush and cluster protocol). Although proven equally effective,^22^ purified aqueous preparations resulted in fewer systemic side effects and smaller local reactions in a comparative study, compared to non-purified preparations under the same rush protocol for bee venom immunotherapy.^23^

The aim of this study, therefore, is to evaluate the safety of bee and vespid VIT during the build-up phase in a large study population in Germany by comparing rush and ultra-rush dosing protocols using purified and non-purified venom preparations. We analyzed adverse events and identified potential risk factors associated with severe anaphylactic reactions.

## Patients and methods

### Patient data and cohorts

We retrospectively evaluated the data of 558 patients with honey bee (*Apis mellifera)* and vespid venom (*Vespula vulgaris* and *Vespula germanica)* allergy who were treated with either a rush or ultra-rush protocol in the Department of Dermatology, Allergology and Venereology from 2005 to 2018. Written informed consent was obtained from the patients for allergological work-up and initiation of VIT. The study protocol for retrospective data collection was approved by the Ethical Board of the University.

Patients were selected for VIT based on the criteria established by the European Academy of Allergy and Clinical Immunology (EAACI).^4^ Immunotherapy was performed in adults and children above the approved age of 9 years, with a history of an immediate type systemic reaction grade ≥1 according to the *Ring and Meßmer*^10^ following a Hymenoptera sting and demonstration of IgE-mediated serum antibodies and/or a positive skin test (prick and/or intradermal skin test) to the respective venom.

From 2005 to 2012, patients were treated using a rush protocol, since 2012 an ultra-rush protocol was routinely used at our center. We retrospectively analyzed the rush and the ultra-rush protocol group as well as the honeybee and vespid VIT group.

### Allergological testing

Prick and/or intradermal skin testing with Hymenoptera venoms (ALK-Abelló, HØrsholm, Denmark) was performed in accordance with international guidelines.^24^ Total serum IgE and venom-specific serum IgE at the time of VIT initiation was measured using the ImmunoCAP™ method (Thermo Fisher Scientific, Freiburg, Germany). Results >0,35 kU/L were considered positive.

### Venom immunotherapy

VIT was performed at our inpatient clinic with aqueous honeybee or vespid venom extract containing 100 μg allergen per ml. The purified preparations ALK-lyophilized bee venom SQ® 801 and ALK-lyophilized vespid venom SQ® 802 (ALK-Abelló) were administered in the ultra-rush cohort and the non-purified Reless® honeybee venom and Reless® vespid venom (ALK-Abelló) were used in the rush cohort. Unless indispensable, ACE-inhibitors and betablockers were paused or switched to an alternative drug for the duration of the build-up phase in all patients before treatment. The venom maintenance dose of 100 μg was achieved using standardized 2-day ultra-rush and 5-day rush induction protocols. Table 1, Table 2 show the detailed dosing scheme of both protocols. Patients were discharged after a monitoring phase of 24 h after the last injection.

**Table 1:** Two-day ultra-rush protocol dosing scheme. Daily cumulative dose 151.11 μg (day 1) and 200 μg (day 2)

Day	Injection Number	Concentration (SQ units/mL)	Injection volume (mL)	Venom concentration (μg)
1	1	100	0.1	0.01
	2	1.000	0.1	0.1
	3	10.000	0.1	1
	4	100.000	0.1	10
	5	100.000	0.2	20
	6	100.000	0.4	40
	7	100.000	0.8	80
2	8	100.000	1.0	100
	9	100.000	1.0	100

**Table 2:** Five-day rush protocol dosing scheme. Daily cumulative dose 0.34 μg (day 1) and 7.2 μg (day 2), 78 μg (day 3), 180 μg (day 4) and 180 μg (day 5)

Day	Injection Number	Concentration (μg/mL)	Injection volume (mL)	Venom concentration (μg)
1	1	0.1	0.2	0.02
	2	0.1	0.4	0.04
	3	0.1	0.8	0.08
	4	1	0.2	0.2
2	5	1	0.4	0.4
	6	1	0.8	0.8
	7	10	0.2	2
	8	10	0.4	4
3	9	10	0.8	8
	10	10	1.0	10
	11	100	0.2	20
	12	100	0.4	40
4	13	100	0.4	40
	14	100	0.6	60
	15	100	0.8	80
5	17	100	0.8	80
	18	100	1.0	100

In the event of objective anaphylactic reactions during the build-up phase, patients received treatment with oral or intravenous antihistamines and/or corticosteroids. In case of anaphylactic reactions, the dosing protocol was modified by reducing the venom dose or discontinuation of VIT. It is customary to decrease the patient's dose after an SR.^25^ Dose adjustment schedules are highly variable. In our patients with mild systemic reactions as urticaria and angioedema, the dose was reduced to previously tolerated or decreased to approximately 50%. Furthermore the remaining injection intervals were extended from 30 min to 60–120 min.

In the case of dose reduction sometimes the build-up phase was prolonged by additional treatment days until the maintenance dose was reached.

### Predictors of tolerability

Potential predictors of tolerability during VIT induction therapy were defined as dose-protocol (ultra-rush or rush), venom (bee or wasp), age, gender, elevated serum levels of total and specific IgE antibodies, and severity of index reaction according to the *Ring and Meßmer* classification.

### Data collection

In total, we analyzed 581 immunotherapy induction therapies in 558 patients. Clinical data, results of allergological testing and information on VIT-induced adverse events, antiallergic treatment, and protocol modification or discontinuation were retrieved from patient records using the digital hospital information system ORBIS (AGFA HealthCare GmbH, Bonn, Germany). Some patients were considered twice in the evaluation due to double immunotherapy in patients allergic to both bee and vespid venom.

### Statistical analysis

Statistical analysis was performed with SPSS version 23 for Windows (SPSS Inc., Chicago, Illinois, USA). In most of our testing the Chi square or, if applicable, Fisher's exact test were conducted for ordinal and categorical data. The odds ratio (OR) was then calculated for variables that showed a significant difference between cohorts. In addition, for comparison of the venom cohorts, Crammers V was calculated. A binary logistic regression model was used to estimate the probability of VIT-induced urticaria (anaphylaxis grade 1), venom dose reduction and antiallergic therapy in relation to several predictor variables (dose protocol, venom, age, gender, baseline serum total and specific IgE antibody levels as well as severity of index reaction according to the *Ring and Meßmer* classification). Two-sample *t*-test was performed for determination of significant differences in serum total and specific IgE levels and anaphylactic reactions in the respective cohorts. *P* values < 0,05 were considered statistically significant.

## Results

### Patient characteristics

A total of 558 patients between the ages of 9 and 82 years (236 males [42.3%], 322 females [57.7%]) receiving induction VIT for Hymenoptera venom allergy between 2005 and 2018 were included in our study. Table 3 shows patient characteristics at baseline in the rush and ultra-rush cohort.

**Table 3:** Clinical patient data

	Total	Rush protocol	Ultra-rush protocol
**No. of patients**	n = 558	240 (43.0%)	318 (57.0%)
**Allergy**	n = 558	n = 240	n = 318
Honeybee venom	80 (14.3%)	33 (13.8%)	47 (14.8%)
Vespid venom	478 (85.7%)	207 (86.3%)	271 (85.2%)
**Age (y)**			
Range	9–82	9–82	13–79
Mean	46.2 (±14.6)	45.9 (±15.5)	46.3 (±14.1)
**Gender**			
Male	236 (42.3%)	112 (46.7%)	124 (39.0%)
Female	322 (57.7%)	128 (53.3%)	194 (61.0%)
**Grade of index sting reaction**	n = 553	n = 236	n = 317
Grade 1	132 (23.9%)	64 (27.1%)	68 (21.5%)
Grade 2	303 (54.8%)	125 (53.0%)	178 (56.2%)
Grade 3	112 (20.3%)	42 (17.8%)	70 (22.1%)
Grade 4	6 (1.1%)	5 (2.1%)	1 (0.3%)

### Safety of venom immunotherapy: ultra-rush vs. rush protocol cohort

Due to double immunotherapy a total of 581 build-up phases were included in our study. 325 (55.9%) induction treatments followed the ultra-rush protocol and 256 (44.1%) the rush protocol. Table 4 depicts the results of our analysis.

**Table 4:** Safety and protocol modifications of venom immunotherapy. ∗Chi-Square test or Fisher's exact test

	Rush protocol	Ultra-rush protocol	*p-value*	Honeybee venom	Vespid venom	*p-value*
**Total No. of VIT**	256 (44.1%)	325 (55.9%)		82 (14.1%)	498 (85.9%)	
**Protocol deviation**						
None	251 (99.2%)	314 (97.2%)	*0.055*	78 (96.9%)	486 (98.2%)	*0.231*
Delay	2 (0.8%)	9 (2.8%)		3 (3.7%)	9 (1.8%)	
**Dose reduction**	3 (1.2%)	14 (4.3%)	***0.026***	3 (3.7%)	14 (2.8%)	*0.721*
**Therapy discontinuation**	3 (1.2%)	2 (0.6%)	*0.659*	1 (1.2%)	4 (0.8%)	*0.535*
**Urticaria**	8 (3.1%)	26 (8%)	***0.013***	8 (9.8%)	26 (5.2%)	*0.124*
**Anaphylactic reactions**						
no grade 2–4	249 (97.3%)	312 (96%)	*0.105*	78 (95.1%)	482 (96.8%)	*0.517*
grade 2	5 (2%)	13 (4%)		4 (4.9%)	4 (2.8%)	
grade 3	2 (0.8%)	0 (0%)		0 (0%)	2 (0.4%)	
grade 4	0 (0%)	0 (0%)		0 (0%)	0 (0%)	
**Subjective symptoms**	10 (3.9%)	11 (3.4%)	*0.738*	6 (7.3%)	15 (3%)	*0.100*
**Antiallergic therapy**						
Antihistamines	15 (5.9%)	32 (9.8%)	*0.080*	9 (11%)	38 (7.6%)	*0.304*
Corticosteroids	2 (0.8%)	6 (1.8%)	*0.476*	2 (2.4%)	6 (1.2%)	*0.315*

Three hundred fourteen VIT inductions using the ultra-rush protocol were completed within the regular duration of 2 days (97.2%). In the rush protocol cohort, 251 (99.2%) VIT inductions were performed within the regular duration of 5 days (Table 4).

In the ultra-rush protocol cohort, urticaria as a mild systemic anaphylactic reaction during the induction phase was reported in 26 VIT (8.0%), in the rush protocol cohort only 8 incidents of urticaria (3.1%) were noted. Therefore, urticaria occurred significantly more often when using the ultra-rush protocol (p = 0.013, OR 2.70, 95% Confidence Interval (CI) 1.20–6.06) (Table 4).

During the build-up phase of VIT using the ultra-rush protocol, there was no significant difference between the rush and ultra-rush protocol group concerning grade 2 to 4 anaphylactic reactions (p = 0.105), subjective symptoms including headache, vertigo and fatigue (p = 0.738), administration of antihistamines (p = 0.080) or corticosteroids (p = 0.476), the rate of VIT discontinuation (p = 0.659) and protocol time deviation in reaching the maintenance dose (p = 0.055) (Table 4). Intramuscular epinephrine injections were not needed. Detailed data on systemic reactions are presented in Table 5.

**Table 5:** Patient's data on anaphylactic reactions grade 2 to 4. BV, bee venom; f, female; m, male; R, rush; UR, ultra-rush; VV, vespid venom

Patient	Protocol	Venom	Age	Sex	Grade	Presenting Symptoms	Venom concentration (μg)
37	UR	BV	50	f	2	dyspnea	100
532	UR	BV	28	f	2	dyspnea, hypotension, tachycardia	100
537	UR	BV	59	f	2	pruritus, nausea	80
577	UR	BV	53	f	2	urticaria, hypotension, tachycardia	100
55	UR	VV	73	f	2	dyspnea	10
101	UR	VV	46	m	2	nausea, diarrhea	100
179	UR	VV	41	f	2	flush, urticaria, nausea	80
231	UR	VV	67	m	2	hypotension, tachycardia, dyspnea	100
348	UR	VV	35	f	2	pruritus, hypotension, tachycardia	80
359	UR	VV	43	f	2	nausea, vomiting	40
400	UR	VV	43	f	2	urticaria, dyspnea	80
465	UR	VV	51	f	2	hypotension, tachycardia	80
489	UR	VV	47	f	2	flush, hypotension, tachycardia	1
247	R	BV	43	m	2	urticaria, hypotension, tachycardia	80
2	R	VV	40	f	3	hypotension, tachycardia, severe dyspnea	60
8	R	VV	36	f	3	flush, nausea, vomiting, hypotension, tachycardia	0,4
76	R	VV	23	f	2	flush, hypotension, tachycardia	40
196	R	VV	33	m	2	hypotension, tachycardia	100
219	R	VV	45	f	2	dyspnea	10
339	R	VV	41	f	2	nausea, hypotension, tachycardia	80

In the ultra-rush group, in 14 VIT (4.3%) dose reduction was required during the build-up phase, and in the rush group in 3 VIT (1.2%) a modification of the protocol was performed. The frequency of dose modification, therefore, was significantly higher in ultra-rush group (p = 0.026, OR 3.80 95% CI 1.08–13.4) (Table 4).

### Safety of venom immunotherapy: honeybee vs. vespid venom cohort

498 (85.9%) vespid and 82 honeybee (14.1%) VIT inductions were performed, with 1 missing data due to lack of documentation (Table 3). In the bee venom cohort, 8 cases (9.8%) of urticaria and in the vespid venom group 26 cases (5.2%) of urticaria were reported. No statistically significant difference was found between both cohorts concerning the incidence of urticaria (p = 0.124), grade 2 to 4 anaphylactic reactions (p = 0.517), subjective symptoms (p = 0,100), antiallergic treatment with antihistamines (p = 0.304) or corticosteroids (p = 0.315), therapy discontinuation (p = 0.535), dose reductions (p = 0.721) and protocol deviation (p = 0.231) during build-up phase. The results above are displayed in Table 4 for better overview.

### Predictors of tolerability

Graphical analysis and Shapiro-Wilk test showed a significant Gaussian normal distribution of total IgE levels and specific IgE against bee and vespid venom (p = 0.395). We applied the two-sample *t*-test and found no statistically significant difference concerning the mean value of total serum IgE levels nor specific IgE against bee and vespid venom in the following side effect cohorts: systemic reactions grades 2 to 4, subjective symptoms or urticaria during VIT initiation, need for antihistamine or corticosteroid treatment, dose reduction or discontinuation of therapy, as well as attainment or non-attainment of the maintenance dose during VIT induction phase. We did not find any significant differences between these subcohorts regarding total or specific IgE values.

Baseline tryptase concentrations (BTCs) were determined in 262 VITs (45.1%). A basal tryptase value of <11.4 μg/mL was considered normal. 12 cases were above the threshold value > 11.4 μg/mL (4.8%). In the elevated BTC cohort, grade II-IV anaphylaxis occurred in 3 VITs (25.0%), in the normative BTC group in 7 VITs (2.8%). The frequency of grade II-IV anaphylaxis, therefore, was significantly higher in the elevated BTC group (OR 11.5714; 95%-KI: 2.5631–52.2401; p = 0.007). There was no significant difference in the occurrence of urticaria between the two groups (OR 1.5325; 95%-KI: 0.1845–12.7284; p = 0.515).

Our binary logistic regression model demonstrated that the ultra-rush protocol is a significant predictor of our dependent variable urticaria (p = 0.017; OR = 2.68). Urticaria was also 1.96-times more frequent in bee venom VIT, this association, however, was not statistically significant (p = 0.116; OR: 1.96). We were not able to identify any further predictors of urticaria during VIT induction. In our model total serum IgE levels, specific serum IgE levels, age, gender, and severity of index reaction according to the *Ring and Meβmer* classification were no significant predictors for occurrence of urticaria, performance of dose reduction or use of antihistamines or corticosteroids for the treatment of VIT-related adverse effects.

## Discussion

Venom immunotherapy is generally considered a safe and effective treatment to prevent potentially life-threatening reactions in Hymenoptera venom allergic patients. The frequency of systemic adverse events with VIT in large multi-center studies ranges from 8 to 20%.^11^, ^12^, ^13^, ^14^ Most studies, however, are based on highly variable study designs and small numbers of patients resulting in conflicting data. Until today, controversy regarding the optimal dosing protocol, therefore, remains. The aim of this retrospective observational study was to evaluate the safety and tolerability of a rush and ultra-rush protocol in a large cohort of 581 immunotherapy induction therapies in patients with honeybee and vespid venom allergy.

### Rush vs. ultra-rush protocol

The frequency of systemic reactions for ultra-rush protocols reported in previously published studies ranges from 0% to 30%^21^^,^^26^, ^27^, ^28^ and for rush protocols from 10% to 17.9%.^13^^,^^15^^,^^16^^,^^20^^,^^29^ Rapid dose increase during the build-up phase has previously been established as a risk factor for systemic reactions.^11^^,^^12^ In our study, the risk of developing urticaria was 2.7 times higher in the ultra-rush protocol cohort compared to the rush protocol cohort (8% vs 3.1%, p = 0.013), which is higher than previously reported by Brehler et al (5.2% vs. 4.2%).^15^

These findings support the premise, that mild anaphylactic reactions occur more frequently when following the ultra-rush protocol. The difference in frequency of occurrence of anaphylactic reactions grade 2 to 4, on the other hand, was not significant between the two dosing protocols (p = 0.105). Severe (grade 4 anaphylaxis according to *Ring and Meßmer*) or lethal adverse events did not occur in our study population. Wenzel et al reported considerably higher rates of severe side effects with grade 4 anaphylactic reactions (*Müller* classification) occurring in 2.2% of VIT following a rush-protocol.^16^

Frequency of subjective symptoms such as headache, vertigo, fatigue, and flush, was similar in both cohorts (p = 0.100). These symptoms are also observed in Hymenoptera field stings, and, therefore, can be expected to occur during VIT as a result of immunological stimulation. Psychogenic causes may also possibly contribute to a wide spectrum of subjective symptoms. Furthermore, anxiety-related symptoms, which are difficult to objectify, may lead to an overestimation of anaphylaxis rates and possibly explains inconsistent data on the frequency of systemic reactions.^30^

Dose reductions due to side effects were significantly more frequent in the ultra-rush protocol cohort, (4.3% vs 1.2%; p = 0.026). The rate of dose modification using the ultra-rush protocol is comparable to the findings of Brehler et al (6.6%).^15^ Hence, adverse events requiring dose modification can be expected to occur more frequently using the ultra-rush protocol.

The use of antihistamines and corticosteroids also serves as an indicator for systemic anaphylactic reactions or large local reactions during VIT. In the ultra-rush protocol cohort intervention with antihistamines and corticosteroids occurred more often compared to the rush protocol cohort (9.8% vs 5.9% for antihistamines, p = 0.080 and 1.8% vs 0.8% for corticosteroids, p = 0.476). Although the increase was not statistically significant, this trend also indicates that side effects requiring antiallergic-treatment are more frequent when performing an ultra-rush VIT.

Despite the reported side effects, the rate of discontinuation of therapy, was very low in both cohorts (1.2% in the rush vs 0.6% in the ultra-rush protocol cohort) in comparison to other studies reporting discontinuation rates of up to 4.9% in rush protocol cohorts^16^ and 4% in ultra-rush cohorts.^27^ The comparison of different studies is impeded, however, due to different classifications for grading of anaphylactic reactions and different allergen preparations in use, which may explain the wide deviation of results.

Abiding to the European drug approval status at the time, in our study we used 2 different allergen solutions of the same manufacturer (ALK-Abelló): Since 2012 ALK-lyophilized SQ® 801 and SQ® 802 for the ultra-rush and before 2012 Reless® honeybee and vespid venom for the rush protocol. Both preparations present aqueous solutions containing the same allergen amount, but ALK-lyophilized SQ® 801 and 802 solutions are raw preparations purified from peptides and active amine components, which are associated with local reactions.

In Europe, both purified and non-purified venom extracts containing 100 μg/mL of allergens are used for VIT. Purified venom extracts contain no low molecular components such as vasoactive amines (dopamine, histamine, and serotonin) and only reduced concentrations of small peptides (apamine, kinine, and mast cell degranulation peptide) present in native venom extract (cut-off: 1000 D).^23^ Comparative studies have shown that purified and non-purified venom extracts show comparable efficacy in terms of tolerance induction.^23^ Purified aqueous venom extracts, however, appear to be safer than non-purified preparations, in particular due to significantly lower rates of large local reactions.^23^ Biló et al showed a lower rate of systemic reactions during build-up phase when using purified aqueous preparations compared to non-purified preparation in a study with honeybee VIT.^23^ Nittner-Marszalska et al, on the other hand, reported no difference in terms of systemic side effects between both preparations.^29^

In our study, the use of different allergen solutions may have hampered statistical analysis. A higher purification grade, however, was not associated with improved tolerability, as urticaria occurred more frequently in the ultra-rush cohort using purified solutions and grade 2–4 reactions were equally distributed in both cohorts. The choice of allergen preparation, thus, seems to have less influence on tolerability than the dosing scheme used during VIT initiation. On the other hand, the positive effect of a higher purification grade on tolerability may have been antagonized by the expeditious protocol in the ultra-rush group.

Rapid induction with early attainment of the maintenance dose is associated with a reduced risk of anaphylaxis in the event of Hymenoptera stings during immunotherapy^4^^,^^16^^,^^26^ and leads to high acceptance of rapid induction protocols among patients.^26^ Both the ultra-rush and rush protocol allow for prompt attainment of the maintenance dose (99.4% in the ultra-rush vs 98.8% in the rush protocol cohort, p = 0.695). Although dose reductions and protocol modifications were more frequent in the ultra-rush cohort, the duration of the build-up phase in the ultra-rush cohort was delayed only by a maximum of 2 additional days leading to an overall faster attainment of the maintenance dose compared to the rush-protocol with a minimum duration of 5 days (Table 4). The ultra-rush protocol, therefore enables faster venom tolerance requiring less injections and thereby facilitates shorter patient hospitalization and improves patient adherence.^31^^,^^32^

The top objective of the build-up phase is attaining the maintenance dose without causing any reactions. Research has revealed that, up to 10% of the time, shorter, one-day, ultra-rush protocols that afford patients same-day discharge have caused severe systemic reactions that require treatment with adrenaline.^27^ Beyond that, the maintenance dose is typically achieved in only 88.4% of the patients.^27^

Economic reasons also do not justify discharging patients on the same day of dosing, which can endanger the patient's safety and risk the psychological burden of suffering another severe iatrogenic anaphylaxis.

Especially delayed and/or biphasic systemic reactions, which can occur in up to 23% of VIT,^33^^,^^34^ are not considered in these one day-protocols.^27^^,^^28^

Premedication with Histamine H1 receptor Antagonists has been shown to be safe and effective^35^^,^^36^ and is often performed routinely during VIT induction in order to increase tolerability and reduce local and systemic side effects. Since allergic reactions are seen in only a small proportion of patients, at our center we only use antihistamines therapeutically in the event of side effects.

### Bee venom vs vespid venom

A number of studies, consistently described bee venom as a major risk factor^4^^,^^13^^,^^21^^,^^36^ with a 3.1- to sixfold higher risk for systemic adverse events during VIT.^17^^,^^37^^,^^38^ Possible explanations include considerably higher venom amounts injected during a honeybee field sting than during vespid stings. During honeybee VIT build-up, therefore, more injections with subclinical antigen amounts are administered, promoting proallergic mechanisms by stimulation of high-affinity IgE receptors in regulatory T-cells.^11^ Another reason may be that vespid venom is obtained from the venom sac and thus includes proteases that can degrade vespid venom allergens, thereby making it potentially less allergenic.^39^

In our study, independently of the induction protocol used, we found no statistically significant difference concerning the tolerability of immunotherapy between the bee and vespid venom cohort. Our results indicate a trend, however, towards a higher incidence of adverse reactions in patients receiving bee venom extract.

Risk of urticaria (9.8% vs 5.3%, p = 0.124) and general symptoms (7.3% vs. 3.0% p = 0.100) was higher in the bee than in the vespid venom cohort, resulting in a higher frequency of antihistamine (7.3% vs 3.0%, p = 0.304) and corticosteroid administration (2.4% vs 1.2%, p = 0.315). The rate of grade 2 to 4 systemic reactions was comparable in both group (p = 0.517). The rate of dose reductions (3.7% vs 2.8%, p = 0.721) and of prolongation of the build-up phase was also higher in the bee venom group (3.7% vs 1.8%, p = 0.231). These results are consistent with previous studies.^28^^,^^40^

### Predictors of tolerability

Our results support prior findings^4^^,^^11^^,^^13^^,^^28^^,^^41^ in demonstrating that total serum IgE and specific IgE levels, age, gender, and severe initial sting reactions do not correlate with higher rates of adverse events or protocol modifications.

Regardless of the build-up phase and insect venom, the cohort with increased BTCs had significantly more grade 2 to 4 anaphylaxis. Mastocytosis and BTCs exceeding 20 μg/mL are associated with a higher risk of adverse events in VIT for a vespid allergy.^4^ Whether a moderately increased BTCs (>11.4 μg/mL < 20.0 μg/mL) can be regarded as a general risk factor has also been debated.^4^^,^^11^^,^^42^^,^^43^ At the same time, our data on BTCs need to be interpreted with caution due to the small amount of elevated BTCs. In fact, whether the build-up protocol or insect venom may have influenced this cohort cannot be deduced from the data due to the low number of cases.

The frequency of urticaria in our patients did not correlate with elevated BTCs, which aligns with recently published data showing that the absence of urticaria and angioedema can be regarded as predictors of severe anaphylaxis associated with elevated BTC.^44^, ^45^, ^46^

Most previous studies are consistently stating that VIT is reasonable safe and severe side effect are rare.^11^ But regarding potential predictors of tolerability and risk factors of VIT current literature is inconsistent. A major limitation of most available data including ours, is the retrospective nature of the analysis. Further prospective studies including large patient numbers are needed for identification of predictors of tolerability and stratification of patients at risk for adverse reactions during VIT.

## Conclusion

In conclusion, both rush and ultra-rush VIT induction protocols exhibit a high level of safety and tolerability. Use of the ultra-rush protocol reduced the duration of the initiation phase from 5 to 2 day, while resulting only in a 2.7-fold increased rate of mild anaphylactic reactions (urticaria). More frequent dose reductions and treatment of side effects require inpatient monitoring and treatment. Our results, therefore, emphasize the significance of inpatient care in the field of allergology. The risk for life-threatening, severe systemic reactions was not increased compared to the rush protocol. With the ultra-rush dosing scheme, patient hospitalization can be reduced to a minimum and protection is achieved in shorter time, which improves patient compliance and has a positive socioeconomic impact on cost saving strategies in health care. To the authors, ultra-rush immunotherapy in an inpatient setting can thus serve as first line treatment in Hymenoptera venom allergic patients.

## Funding

The authors received no specific funding for this work.

## Availability of data and materials

All relevant data are within the paper.

## Author contributions

All the signing authors contributed to the clinical work (EV, KA, MM, RK), data collection (RS, EV, KA) and analysis of the data and drafting the manuscript (RS, EV, HA, TF, MM, RK, NZ). EV and HA designed the study. HA was the biostatistician.

## Ethics approval

Study protocol was approved for retrospective data collection. The described treatments were part of the standard of care in this field.

## Consent of publication

All authors approved the final version and its submission.

## Conflict of interest disclosure

RS, TF, KA, NZ, HA, RK and MM certify, that they have NO affiliations with or involvement in any organization or entity with any financial interest or non-financial interest in the subject matter or materials discussed in this manuscript.

EV declares intermittent advisory board relationship with ALK-Abelló.
